# Prognostic significance of postoperative serum carcinoembryonic antigen levels in patients with completely resected pathological-stage I non-small cell lung cancer

**DOI:** 10.1186/1749-8090-8-106

**Published:** 2013-04-22

**Authors:** Yoshiki Kozu, Tomohiro Maniwa, Shoji Takahashi, Mitsuhiro Isaka, Yasuhisa Ohde, Takashi Nakajima

**Affiliations:** 1 Division of Thoracic Surgery, Shizuoka Cancer Center, Shimonagakubo 1007, Nagaizumi, Shizuoka, 411-8777, Japan; 2 Division of Pathology, Shizuoka Cancer Center, Shizuoka, Japan

**Keywords:** Non-small cell lung cancer, Unfavorable prognostic factor, Pathological-stage I, Postoperative carcinoembryonic antigen level, Adjuvant chemotherapy

## Abstract

**Background:**

Until date, there are no clear recommendations for regular perioperative measurements of serum CEA levels for lung cancer in any guidelines. The purpose in the present study is to evaluate the prognostic significance of perioperative serum carcinoembryonic antigen (CEA) levels in patients with pathological-stage I non-small cell lung cancer (NSCLC).

**Methods:**

We retrospectively reviewed 263 completely resected pathological-stage I NSCLC patients whose preoperative and postoperative serum CEA levels were measured. Patients were subdivided according to the perioperative change of CEA levels: continuously normal CEA levels (NN group), continuously high CEA levels (HH group), and high preoperative CEA levels that returned to normal levels post-operation (HN group). The clinicopathological factors and overall survival (OS) among these 3 groups were compared. Univariate and multivariate analyses of the correlation between clinicopathological factors and OS were performed.

**Results:**

High preoperative CEA levels significantly correlated with men aged >70 years with smoking history, high serum CYFRA 21–1 levels, greater tumor diameter, presence of visceral pleural invasion (VPI), and moderate-to-poor differentiation. Five-year OS rates in the NN and HH groups were 95.5% and 59.3%, respectively. Four-year OS rate in the HN group was 85.5%. Multivariate analyses indicated tumor diameter of more than 30 mm, presence of VPI, and the HH group were independent unfavorable prognostic factors.

**Conclusions:**

A high postoperative CEA level was an independent unfavorable prognostic factor in pathological-stage I NSCLC patients. Patients with high postoperative CEA levels may benefit from adjuvant chemotherapy.

## Background

In many clinical practices, serum tumor markers are easily and reproducibly evaluated. Measurement of tumor markers in patients with malignancies enables us to diagnose, quantitatively assess cancer cells, predict survival, and monitor the effects of treatment.

Carcinoembryonic antigen (CEA) is one of the most commonly used tumor markers in gastrointestinal and lung cancers. Many reports have already described the significant correlation of preoperative CEA levels with prognosis in patients with early-stage non-small cell lung cancer (NSCLC) [1-7]. However, serum CEA levels are known to be affected by the rate of CEA elimination from circulating blood determined by renal function and smoking status [8].

From this perspective, postoperative CEA levels might be a more accurate prognostic factor than preoperative CEA levels, because the former is less affected by preoperative smoking status. Dent et al. initially reported high values of postoperative CEA levels [9]. Subsequent reports demonstrated that high postoperative CEA levels predicted recurrence and prognosis in NSCLC patients [4,8,10-15]. Until date, however, there are no clear recommendations for regular perioperative measurements of serum CEA levels for lung cancer in any guidelines.

We conducted a retrospective study based on a single institution to evaluate the prognostic importance of perioperative serum CEA levels in patients with completely resected pathological-stage I NSCLC.

## Methods

Between September 2002 and December 2009, 545 patients underwent complete surgical resection for pathological-stage I NSCLC at the Shizuoka Cancer Center Hospital, Shizuoka, Japan. Of these, 263 consecutive patients who met all of the following criteria were included and retrospectively reviewed in the present study: (1) those who had their serum CEA concentrations measured within the 1-month period before surgery and again within the 2-month period after surgery (while preoperative serum CEA levels were measured routinely, the measurement of postoperative CEA levels was at the discretion of the attending surgeon), (2) those without multiple lung tumors or malignancies in other organs and (3) those who were anticancer treatment-naïve in both the neoadjuvant setting and adjuvant setting. Approval from the institutional ethics committee was obtained.

The medical records of each patient were examined for age, gender, smoking history, preoperative European Cooperative Oncology Group performance status (ECOG PS), both preoperative and postoperative serum CEA levels, preoperative serum cytokeratin 19 fragment (CYFRA21-1) levels, tumor location, surgical procedure, tumor histology, tumor diameter, visceral pleural invasion (VPI), angiolymphatic invasion (ALI), differentiation grade, and survival. Staging was determined according to the seventh edition of the International Union Against Cancer (UICC) TNM staging system [16]. Histology was evaluated according to the latest World Health Organization’s classification of lung tumors [17].

We performed lobectomy or pneumonectomy with systemic mediastinal lymph node dissection as the standard surgical treatment. Segmentectomy or wedge resection was performed only on patients with tumors showing a greater proportion of ground-glass opacity on computed tomography or on those with major comorbidities.

Serum CYFRA21-1 concentrations were measured within the 1-month period before surgery (at the same time as CEA). The CEA concentration was measured using an ARCHITECT(r) kit (Abbott Japan, Tokyo, Japan), while the CYFRA21-1 concentration was measured using an Lumipulse Presto(r) kit (FUJIREBIO Inc, Tokyo, Japan). According to the manufacturer of these kits, the upper limits of the percentiles of healthy individuals for CEA and CYFRA21-1 levels were 5.0 ng/mL and 3.5 ng/mL, respectively. Therefore, the cut-off point for these tumor markers was set to the same level as the upper limits in the present study. Patients were subdivided into 3 groups on the basis of their shift of CEA levels by surgery as follows: patients with continuously normal CEA levels (NN group), patients with continuously high CEA levels (HH group), and patients with high preoperative CEA levels that returned to normal levels post-operation (HN group).

ALI was defined as the presence of neoplastic cells within an arterial, venous, or lymphatic lumen during routine histologic evaluation with hematoxylin and eosin, elastic van Gieson (EVG), and D2-40 stains. Because of the retrospective nature of this study, distinction between arterial, venous, or lymphatic invasion were not available in some cases. Therefore, these categories were merged into a single variable (ALI). The presence or absence of ALI was assessed in the largest cross-sections of the tumor in every specimen. VPI was defined as evidence of penetration of the thick outer elastic lamina by the tumor during EVG staining. The grade of differentiation was categorized into well differentiated, moderately differentiated, and poorly differentiated carcinomas according to the degree of structural and cytologic atypia. Differentiation in squamous cell carcinoma was determined on the basis of the degree of keratinization and the presence of intercellular bridges. Poor differentiation in squamous cell carcinoma was defined as a tumor with faint these features and a solid pattern. Adenocarcinoma, which is largely composed of malignant glandular epithelium, was evaluated by lepidic, tubular and papillary structure. Poor differentiation in adenocarcinoma was defined as a solid pattern tumor without any clear gland formation. Pathological evaluation was performed prospectively by 2 or more experienced pathologists.

The clinicopathological factors of the patients (n = 263) are listed in Table 1. There were 132 men and 131 women, with a median age at surgery of 67 years (range, 20–88 years). There were 198, 44, and 21 patients in the NN, HN, and HH groups, respectively. Pathological examinations revealed that there were 213 adenocarcinomas, 46 squamous cell carcinomas, 2 adenosquamous carcinomas, and 2 carcinoids.

**Table 1:** Clinicopathological factors of the total study population

**Factors**	**Total**	**NN group**	**HN group**	**HH group**	***p*** **value** ^**a**^
	**(n = 263)**	**(n = 198)**	**(n = 44)**	**(n = 21)**	
Age					0.010
≤70 y	140	116	16	8	
>70 y	123	82	28	13	
Gender					0.002
Male	132	87	30	15	
Female	131	111	14	6	
Smoking history					< 0.001
≥5 PY	135	85	33	17	
<5 PY	128	113	11	4	
ECOG PS					0.28
0	222	169	38	15	
1	41	29	6	6	
Serum CYFRA					0.039
>3.5 ng/mL	12	5	5	2	
≤3.5 ng/mL	251	193	39	19	
Location					0.77
Right	167	124	30	13	
Left	96	74	14	8	
Surgical procedure					0.98
Sublobar resection	38	29	6	3	
Major resection ^b^	225	169	38	18	
Tumor diameter					0.002
>30 mm	53	30	17	6	
≤30 mm	210	168	27	15	
Histology					0.27
Adeno	213	165	33	15	
Squamous	46	30	11	5	
Others	4	3	0	1	
VPI					0.037
Present	50	31	11	8	
Absent	213	167	33	13	
ALI					0.32
Present	66	49	9	8	
Absent	197	149	35	13	
Differentiation ^c^					< 0.001
Well	168	140	19	9	
Moderate/Poor	91	55	25	11	

*PY*, Pack-years; *ECOG PS*, European cooperative oncology group performance status; *CYFRA*, Cytokeratin 19 fragment; *VPI*, Visceral pleural invasion; *ALI*, Angiolymphatic invasion.

^a^association between the NN group and HN group, the chi-square test.

^b^ Major resection refers to lobectomy or pneumonectomy.

^c^ The grade of differentiation was not determined in 4 patients with adenosquamous carcinoma or carcinoids.

Overall survival (OS) was calculated as the period from surgical resection until death or the date of the last follow-up evaluation. Last actualization of survival data was performed in September 2012. Follow-up was completed in 245 of the patients. The median time for follow-up for surviving patients was 54 months (range, 15–120 months). Of the total, 22 patients (8.4%) died during follow-up. The causes of death were primary disease in 21 patients and comorbid diseases in 1.

### Statistical analysis

Counts were compared using the chi-square test. The Kaplan-Meier method was used to generate survival curves, and survival differences were analyzed using the log-rank test. Multivariate analyses of prognostic factors were performed using the Cox proportional hazards model. A *p*- value of less than 5% was considered significant. All statistical analyses were performed using JMP 9 software (SAS Institute, Cary, NC, USA).

## Results

### Patients’ clinicopathological factors according to the CEA group

The HN group significantly correlated to older men (>70 y) with a smoking history (≥5 pack-years), high serum CYFRA 21–1 levels (>3.5 ng/mL), greater tumor diameter (>30 mm), presence of VPI, and moderate-to-poor differentiation compared to the NN group (Table 1).

### OS curves according to the CEA group

**Figure 1:**
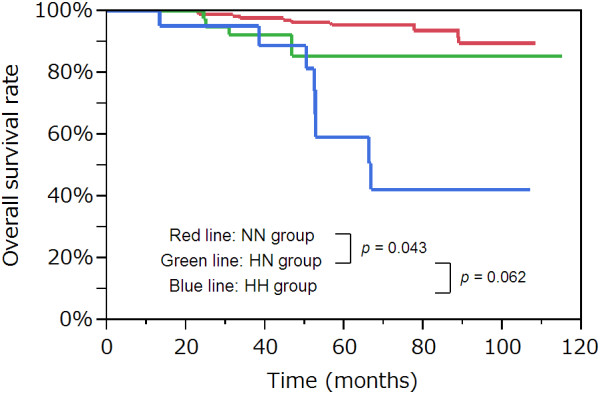
**Overall survival curves according to the CEA group.** A significant difference in overall survival was observed between the HN group and NN group (*p* = 0.043). There was a marginally significant difference in overall survival between the HH group and HN group (*p* = 0.062). CEA, carcinoembryonic antigen.

Five-year OS rates in the NN and HH groups were 95.5% and 59.3%, respectively. Four-year OS rate in the HN group was 85.5%. A significant difference in OS was observed between the HN and NN groups (*p* = 0.043). A trend toward a decreased survival was observed for the HH group compared to the HN group, but was not statistically significant (*p* = 0.062). These survival curves are shown in Figure 1.

### Predictors of OS according to univariate analyses

The results of univariate analyses for survival are summarized in Table 2. The clinicopathological factors of age, ECOG PS, serum CEA levels, tumor diameter, VPI, ALI, and differentiation grade were significant prognostic factors for OS.

**Table 2:** Predictors of OS according to univariate analysis

**Risk factors**	**Favorable**	**Unfavorable**	***p *Value **^**a**^
Age	≤70 y	>70 y	0.008
Gender	Female	Male	0.12
Smoking history	<5 PY	≥5 PY	0.088
ECOG PS	0	1	0.007
Serum CEA	NN group	HN group	0.043
	HN group	HH group	0.062
Serum CYFRA	≤3.5 ng/mL	>3.5 ng/mL	0.90
Location	Left	Right	0.99
Surgical procedure	Major resection ^b^	Sublobar resection	0.57
Tumor diameter	≤30 mm	>30 mm	< 0.001
Histology	Adeno	Others	0.070
VPI	Absent	Present	0.001
ALI	Absent	Present	0.008
Differentiation	Well	Moderate/Poor	0.037

*OS*, Overall survival; *PY*, Pack-years; *ECOG PS*, European cooperative oncology group performance status; *CEA*, Carcinoembryonic antigen; *CYFRA*, Cytokeratin 19 fragment level; *VPI*, Visceral pleural invasion; *ALI*, Angiolymphatic invasion.

^a^ the log-rank test.

^b^ Major resection refers to lobectomy or pneumonectomy.

### Predictors of OS according to multivariate analyses

Multivariate analyses for OS were performed using these factors, and the results are summarized in Table 3. Tumor diameter of more than 30 mm (hazard ratio [HR] = 3.47, 95% confidence interval [CI]: 1.35–8.98, *p* = 0.010), presence of VPI (HR = 3.18, 95% CI: 1.11–8.84, *p* = 0.032), and the HH group (HR = 7.69, 95% CI: 2.46–23.7, *p* < 0.001) were found to be independent unfavorable prognostic factors.

**Table 3:** Predictors of OS according to multivariate analysis

**Risk factors**	**HR**	**95% CI**	***p *value **^**a**^
Age			
≤70 y	1	Reference	
>70 y	2.29	0.87–6.72	0.095
ECOG PS			
0	1	Reference	
1	1.33	0.47–3.53	0.59
Tumor diameter			
≤30 mm	1	Reference	
>30 mm	3.47	1.35–8.98	0.010
VPI			
Absent	1	Reference	
Present	3.18	1.11–8.84	0.032
ALI			
Absent	1	Reference	
Present	1.06	0.38–3.15	0.91
Differentiation			
Well	1	Reference	
Moderate/Poor	1.14	0.42–3.01	0.80
Serum CEA			
NN group	1	Reference	
HN group	2.52	0.72–8.11	0.14
HH group	7.69	2.46–23.7	< 0.001

*OS*, Overall survival; *HR*, Hazard ratio; *CI*, Confidence interval; *ECOG PS*, European cooperative oncology group performance status; *VPI*, Visceral pleural invasion; *ALI*, Angiolymphatic invasion; *CEA*, Carcinoembryonic antigen.

^a^ the log-rank test.

### OS curves according to the number of unfavorable prognostic factors

**Figure 2:**
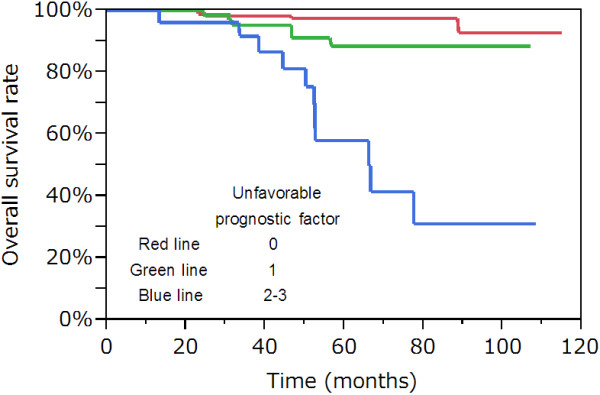
**Overall survival curves according to the number of independent unfavorable prognostic factors (tumor diameter of more than 30 mm, presence of VPI, and the HH group) on the basis of the results of multivariate analyses.** Five-year overall survival rate was 58.0% for those harboring 2 or more unfavorable prognostic factors (n = 25). VPI, visceral pleural invasion.

Figure 2 shows OS curves according to the number of independent unfavorable prognostic factors (tumor diameter of more than 30 mm, presence of VPI, and the HH group) on the basis of the results of multivariate analyses. Five-year OS rate was 58.0% for those harboring 2 or more unfavorable prognostic factors (n = 25).

## Discussion

There have been 3 reports published, which were similar in design to the present study [4,11,15] and subdivided pathological-stage I NSCLC patients into 3 groups, viz. the NN, HN, and HH groups (Table 4). These previous reports have demonstrated that the worst prognosis correlates to the HH group, consistent with results of the present study. Taken together, these results suggest that failure to normalize CEA levels after surgery is associated with a significantly worse prognosis. Okada et al. speculated that failure to achieve normal levels of CEA levels is caused either by unrecognized extrapulmonary disease or failure to eradicate all pulmonary diseases [4]. The results in the current study show that the 5-year OS rate in the HH group was 59.3%, the highest compared to those in previous reports (range, 36.0%–48.6%). A similar trend was observed among the HN and NN groups as well. The previous reports included incomplete resections [4] and higher percentages of pathological-stage IB patients (60.3% vs. 33.0% compared with the present study) [15], and these differences could be the possible reason for decreased survival.

**Table 4:** Comparison of the previous reports analyzing the perioperative change of serum CEA levels in patients with pathological stage-I NSCLC

**Author**		**Okada **[4]	**Matsuguma **[11]	**Wang **[15]	**Present study**
Year		2004	2008	2010	2013
CEA measurement period	Before surgery	Within 1 M	Within 1 M	Within 2 W	Within 1 M
	After surgery	Within 1 M	Within 1–3 M	Within 1–3 M	Within 2 M
Cut-off point (ng/mL)		5.0	5.0	6.0	5.0
Total number		722 ^a^	455	257	263
NN group	(5-ys)	472 (84.2%)	323 (85.9%)	173 (71.1%)	198 (95.5%)
HN group		154 (74.2%)	112 (56.2%)	56 (54.6%)	44 (85.5%) ^b^
HH group		96 (48.6%)	20 (43.1%)	28 (36.0%)	21 (59.3%)
		Independent prognostic factor		N/A	Age	Age	Tumor diameter
	Histology				HH group	VPI
	VPI					HH group
	HN/HH group					

*CEA*, Carcinoembryonal antigen; *NSCLC*, Non-small cell lung cancer; *M*, Month; *W*, Week; *N/A*, Not analyzed; *VPI*, Visceral pleural invasion.

^a^ including incomplete resections.

^b^ 4-ys.

Matsuguma et al. reported that preoperative high CEA levels significantly correlated with smoking-related factors such as increased age, male gender, squamous cell carcinoma, pathological T2 status, vascular invasion, VPI, and moderate-to-poor differentiation (only in adenocarcinomas) [11]. These observations are mostly consistent with those reported in the present study. Wang et al. reported that histologic adenocarcinomas significantly correlated with high preoperative CEA levels, whereas age, smoking status, tumor diameter, VPI, and pathological-stage did not [15]. One of the most notable differences between the current study and previous reports is that we did not observe any significant correlation between CEA levels and histologic type. Although CEA was initially investigated in colon adenocarcinoma, it has been known to be elevated in patients with squamous cell carcinoma of the lung, uterine cervix, and esophagus [18-22]. The correlation of CEA levels and histologic subtype of NSCLC remained still controversial.

Our results indicate that tumor diameter of more than 30 mm, presence of VPI, and the HH group were independent unfavorable prognostic factors. The former 2 factors are the most representative determinants for lung cancers to be categorized as T2a according to the seventh edition of the UICC TNM staging system [16]. Since T2aN0M0 lung cancers are staged as pathological-stage IB, our results confirmed the prognostic value of pathological-stage classification, which is the most widely-used prognosticator in lung cancer.

We also highlighted the prognostic significance of continuously high perioperative CEA levels (HH group), consistent with the results by Wang et al. [15]. Because all of the patients with postoperative high CEA levels had preoperative high CEA levels as well, the results indicate the need for measuring postoperative CEA levels rather than preoperative CEA levels. On the other hand, Matsuguma et al. reported that both HN and HH groups were independent unfavorable prognostic factors [11], indicating the need for measuring preoperative CEA levels. In the Western countries, however, regular perioperative measurement of serum CEA levels in patients with lung cancer is not commonly performed and is considered less convincing than in patients with colorectal cancer [4,23].

In Japan, adjuvant chemotherapy with UFT is recommended in pathological-stage IB NSCLC patients because the occurrence of postoperative death was found to have decreased in the UFT group as compared to the control group in previous studies [24,25]. As mentioned earlier, our results indicate the prognostic significance of discriminating pathological-stage IB from IA, supporting this recommendation. The most important clinical implications emerging from this study is that patients with postoperative high CEA levels are also good candidates for adjuvant chemotherapy in addition to pathological-stage IB patients. Although in-anatomical factors of tumor markers are not reflected in the current TNM staging system, the results of our study indicate that postoperative high CEA levels can predict survival, representing micrometastases or residual tumor cells, which may not be detected by radiological images and pathological examination.

Independent prognostic significance of postoperative high CEA levels is yet to be proved in advanced NSCLC patients. Sawabata et al. speculated that this might be attributable to the stronger prognostic impact of anatomic factors such as lymph node metastases than tumor markers [13]. On the other hand, Fukai et al. reported that an elevated preoperative CEA levels (≥5 ng/mL) was an independent predictor of survival even in pT1-2N1M0 NSCLC patients [26]. These studies therefore suggest that the prognostic significance of high postoperative CEA levels should also be validated in advanced NSCLC patients.

The present study has several limitations: First is its retrospective nature; second, postoperative CEA levels were measured at the discretion of the attending surgeon, adding significant selective bias into the present study; third, there was a small sample size of the HH group (n = 21) and observed events, making it statistically difficult to interpret results.

## Conclusions

A high postoperative CEA level was an independent unfavorable prognostic factor in completely resected pathological-stage I NSCLC patients. Those with postoperative high CEA levels may benefit from adjuvant chemotherapy.

### Consent

Written informed consent was obtained from the patient for publication of this report and any accompanying images.

## Abbreviations

CEA: Carcinoembryonic antigen; NSCLC: Non-small cell lung cancer; ECOG PS: European cooperative oncology group performance status; CYFRA21-1: Cytokeratin 19 fragment; OS: Overall survival; VPI: Visceral pleural invasion; ALI: Angiolymphatic invasion; UICC: International union against cancer; EVG: Elastic van Gieson.

## Competing interests

This work was supported by the National Cancer Research and Development Fund (23-A-18).

## Authors’ contributions

TM conceived of the study, and participated in its design and coordination and helped to draft the manuscript. ST and MI both advised and interpreted of data. YO and TN participated in critical revision of the manuscript. All authors read and approved the final manuscript.
